# Vaccination strategies for different contact patterns: weighing epidemiological against economic outcomes

**DOI:** 10.1007/s10754-024-09384-1

**Published:** 2024-09-24

**Authors:** Rikard Forslid, Mathias Herzing

**Affiliations:** 1 Department of Economics, Stockholm University, and CEPR, Stockholm, Sweden; 2 School of Business, Örebro University, Örebro, Sweden

**Keywords:** Pandemics, Vaccination, SIR-model, COVID-19, D42, D62, H10, I18, L10

## Abstract

The aim of this paper is to shed light on the economic and epidemiological trade-offs that emerge when choosing between different vaccination strategies. For that purpose we employ a setting with three age groups that differ with respect to their fatality rates. The model also accounts for heterogeneity in the transmission rates between and within these age groups. We compare the results for two different contact patterns, in terms of the total number of deceased, the total number of infected, the peak infection rate and the economic gains from different vaccination strategies. We find that fatalities are minimized by first vaccinating the elderly, except when vaccination is slow and the general transmission rate is relatively low. In this case deaths are minimized by first vaccinating the group that is mainly responsible for spreading of the virus. With regard to the other outcome variables it is best to vaccinate the group that drives the pandemic first. A trade-off may therefore emerge between reducing fatalities on the one hand and lowering the number of infected as well as maximizing the economic gains from vaccinations on the other hand.

## Introduction

The rapid development of vaccines against Covid-19 and the quick ramping up of production facilities has been unprecedented. But vaccines also need to be distributed and administered to susceptible individuals. This is an important logistic challenge and also raises the question of who to vaccinate first.^1^ With the exception of front-line health care workers, most countries started with the older and most fragile part of the population. However, there is also the alternative strategy to first vaccinate a younger part of the population that may be the driver of the infection. The logic of such a strategy is to first target groups that are primarily responsible for spreading the virus in order to quickly reduce the number of infected persons. This reduction in the transmission of the infection could in principle result in fewer deaths in the long run also among the elderly.

This paper employs a SIR-model to examine the eonomic and epidemiological consequences of different vaccination strategies.^2^ While most countries have already vaccinated large shares of their population, the question of who to vaccinate first remains important, as the arrival of new virus variants necessitate booster vaccinations. Naturally the emergence of a different virus would also raise the same considerations. Our aim is to shed light on the economic and epidemiological trade-offs that emerge when choosing between different vaccination strategies. As a reference point we use Sweden at the end of 2020 when the first vaccine doses against Covid-19 were distributed. However, the conclusions that we obtain are general and provide some guidance for decision-makers.

In our model the population is divided into three groups. The first group consists of young individuals (below 20 years of age) that are very unlikely to die from the infection. The second group comprises working-age adults (20-59 years old) that have a slightly higher risk of dying. The third group consists of the elderly (60 years and older) who face a considerably higher fatality rate when being infected. To assess the evolution of the pandemic it is crucial to capture differences in the transmission rates between and within these groups. To that end we use the results from two different sources that have collected data on interactions between different age groups. Both are calibrated to ressemble the situation in Sweden at the end of 2020 when mass vaccinations started to be implemented. We analyze outcomes under these different contact patterns in parallel throughout this study.

Our first source of transmission rates within and between different age groups are based on estimates from Wallinga et al. (2006), which use age-specific Dutch data on face-to-face conversations as a proxy for exposure to infectious respiratory-spread agents. Two important features stand out from this data. First, intra-group transmission rates are higher than transmission rates between age groups. Second, transmissions rates between middle-aged adults and the older group are considerably higher than between young individuals and the older group.^3^

Our second source is the Socrates CoMix social contact survey based on online representative panels of adults as well as parents reporting on behalf of one of their children during various phases of the Covid 19 pandemic in over 20 European countries (Verelst et al., 2021).^4^ As there are no data for Sweden in the CoMix project, we use data collected in Denmark between December 22nd, 2020 and April 16th, 2021 (henceforth referred to as CoMix Denmark). Thus, our analysis uses data collected during a phase in the pandemic when restrictions particularly aimed at protecting the elderly were in place.

We use both these sources to highlight how policies regarding restrictions can influence the outcomes of different vaccination strategies. The CoMix Denmark contact patterns are more asymmetric in the sense that contacts are much more concentrated to the youngest group, which therefore is the key driving force of the pandemic. By contrast, when transmission rates are based on Wallinga et al. (2006) the pandemic is mainly driven by the middle-aged group, which is the largest group and has relatively many contacts with all groups.

We analyze six different vaccination strategies for different infection transmission rates as well as different vaccination rates and vaccine efficacy rates. Under these strategies the three population groups are vaccinated in sequence; since there are three groups there are six permutations. We have also considered alternative strategies, e.g. all groups being vaccinated simultaneously at rates in proportion to their share of the total population; none of these alternative strategies generates optimal outcomes in terms of any of the outcome measures that we focus upon. Throughout our analysis we assume that eventually the entire population will become vaccinated. In reality some people do not get vaccinated for health or other reasons. Since it is difficult to make well-founded assessments of these factors, which are anyway likely to differ between countries, we make this simplifying assumption. If more people abstain from vaccinations the effect is similar to employing a vaccine with a lower efficacy, which we consider when examining the sensitivity of our results.^5^

To assess the implications of different vaccination strategies we will focus on the following outcome measures:

(i) *The share of the population that will have deceased on day *730 * after vaccinations have started.* Reducing the number of fatalities is obviously an important objective. The number of days is chosen to be equivalent to two years duration to make it possible to assess the impact also of low vaccination rates.
(ii) *The share of the population that will have been infected two years after vaccinations have started.* Keeping the total number of infected individuals low is also important; many surviving infected individuals have been ill for a long time and some suffer long-term consequences.
(iii) *The peak of the share of infectious individuals.* From a public health perspective it is desirable to keep the maximum number of infected persons low. For example, the number of treated people increased dramatically during the last weeks of 2020 in many countries. It is therefore of interest to analyze which vaccination strategy dampens the peak infection rate most.
(iv) *Economic gains from vaccinations one year after their start.* We limit our analysis of economic consequences to one year, because differences in strategies appear almost entirely during the first year of vaccinations. When asssessing the economic effects we take into account that working hours of non-infected persons are negatively affected by increasing numbers of infectees.

Key parameters in our analysis are the efficacy of the vaccine, which we here define as the share of vaccinated persons that become immune,^6^ the vaccination rate and the general transmission rate, which to varying degrees are policy parameters. The efficacy of the vaccine can be varied in a few discrete steps by the choice among available vaccines against Covid-19. The vaccination rate can be increased by purchasing more doses and improving the implementation of a vaccine program. The general transmission rate can be influenced by the restrictions that a government imposes on the population.

We assume in our base case a vaccine efficacy of 0.9 in line with the reported levels of some of the vaccines against Covid-19 that have been developed by January 2021 when mass vaccinations started. The recovery rate is assumed to be the same across the age groups and equal 0.2 (implying five days of being infectious). However, as mentioned above, we allow for heterogeneity with respect to transmission rates within and between groups as well as age specific fatality rates. In our base case we assume a general transmission rate of 0.25, such that the infection reproduction number is given by $R_{0}=1.25$, which is roughly in line with estimated levels for Sweden in December 2020. To check the sensitivity of our results we consider the effects of changes in the efficacy rate as well as the transmission rates.

Our main results can be summarized as follows. First, the strategy of vaccinating the elderly first minimizes the fatality rates for most parameter value configurations; this is the case when transmission rates are sufficiently high or the vaccination rate is sufficiently high or the efficacy of the vaccine is sufficiently high. However, fatalities will instead be minimized by vaccinating the group that drives the infection, when these conditions do not hold, for instance, if vaccination rates are low. Using the Wallinga et al. (2006) contact patterns, fatalities are minimized by vaccinating the middle-aged group first if vaccination rates are so low that it would take more than 684 days to cover the entire susceptible population. If instead CoMix Denmark contact patterns are used, the number of deaths is minimized by vaccinating the youngest group first when vaccination rates are low, such that it would take more than 252 days to cover the entire population. Hence, when vaccination proceeds slowly it is more important to dampen the spread of the virus to protect the elderly and therefore first target the group which drives the pandemic. This case may be particularly relevant in some developing countries where vaccination rates are slow, although it must be recognized that the numerical results here are based on a calibration of contact patterns between age groups that apply in developed countries. The effects of a lower vaccine efficacy resembles in some ways the effect of a lower vaccination rate. The threshold value for the vaccination rate, above which first vaccinating the elderly minimizes fatalities, decreases in the efficacy rate.

Second, the total number of infected persons (after 2 years) and the peak share of infectees are always minimized by first vaccinating the group that drives the pandemic, i.e. the middle-aged if Wallinga et al. (2006) contact patterns are used and the youngest group when CoMix Denmark contact patterns are used. Hence, the fastest way of eradicating the disease is to first target the age group that is the main transmitter of the virus.

Finally, the economic gains from vaccination are highest when the group that drives the pandemic is vaccinated first. If Wallinga et al. (2006) contact patterns are used, it is straightforward that the middle-aged group, which contributes most economically, gets vaccinated first. By contrast, if CoMix Denmark contact patterns are used, rather than first vaccinating the working-age population, it is economically most beneficial to first focus on the youngest to contain the spread of the disease We also find that there are substantial economic gains from speeding up the vaccination campaign. For instance, using the Wallinga et al. (2006) contact patterns, we obtain an estimate for the gains from a campaign where the vaccination rate is doubled, such that it takes 171 rather than 336 days to vaccinate the entire susceptible population, amounting to 0.42 per cent of GDP. Using the CoMix Denmark contact patterns, the same increase in the vaccination rate, such that it takes 169 rather than 326 days to vaccinate the entire susceptible population, yields economic gains of 0.52 per cent of GDP.

Hence, given that vaccination rates are sufficiently high, we obtain a trade-off between minimizing fatalities and the other three outcome variables when it comes to the order of vaccination. Vaccinating the oldest group first implies less deaths, in particular at higher vaccination rates, but it comes at the cost of a higher share of the population becoming infected and therefore also a smaller economic gain from vaccinations. The largest difference in economic outcomes between vaccinating the elderly first and vaccinating the group that drives the pandemic first appears at intermediate vaccination rates.

There are a couple of highly related papers. Matrajt et al. (2020) analyze the optimal use of vaccine in an epidemiological model calibrated to U.S. demographics with 16 age groups that have different levels of susceptibility. Their main analysis assumes that vaccination has been carried out at the beginning of the simulations. The central result here is that deaths are minimized when vaccinating older people first, if the efficacy of a vaccine is between 10 and 50 per cent, but that it is optimal to switch to vaccinating younger persons first when the efficacy of the vaccine is above 60 per cent and there is enough vaccine to cover roughly half of the population. They also model a vaccination campaign where the entire population is vaccinated in 25, 50 or 101 weeks. Here they find that deaths are minimized by first vaccinating the elderly at low vaccination rates, but that vaccinating both old and young people is optimal at high vaccination rates. By contrast, we find that it is optimal to start with younger groups, given that vaccination is slow and the infection reproduction number is close to one (such that deaths are not minimized by starting with the oldest group first). This different result is likely to be due to the fact that we are using a full matrix of estimated social interactions between age groups, whereby we account for there being relatively little transmission between the youngest and the oldest, most fragile age groups.

Moore et al. (2020) analyze the optimal sequence of Covid 19 vaccination in the UK in terms of deaths and quality adjusted life years. They divide the population into five age groups and use a social contact matrix between the groups based on UK data. They find that it is always optimal to target older age groups first. Three types of vaccine are analyzed: a vaccine that reduces susceptibility, one that reduces the probability of becoming symptomatic, and one that protects against symptoms becoming severe. The bulk of their analysis is based on the assumption that the vaccine can be instantaneously administered, but they also simulate a case where the speed of vaccine deployment is varied. In contrast to Matrajt et al. (2020) and the present study they find that the optimal ordering of age groups is unaffected by the speed of vaccine deployment.

Bartsch et al. (2020) calibrate a model of the spread of SARS-CoV-2 in the U.S. to identify the vaccine efficacy thresholds, above which vaccination could extinguish an ongoing wave of the pandemic across a range of possible scenarios. Contrary to our paper they do not analyze a vaccination campaign or consider a population divided into age groups with different mortality and transmission rates.

Vellodi and Weiss (2020) analyze optimal vaccination in a model without infection dynamics where individuals are randomly matched. Agents differ in exposure vulnerability and they may voluntary chose to self-isolate. They find that it is optimal to first vaccinate individuals with an intermediate risk of severe illness.

Gollier (2021) analyzes the welfare consequences of different vaccination strategies using an epidemiological model, which is calibrated on French data and contains three age groups. In a setting with two countries it is shown that the observed vaccine nationalism, where rich countries piroritize to fully vaccinate their own population before exporting any vaccine, could increase the global death toll by 20 per cent.

Our paper is also related to a publication by Britton et al. (2020) where population heterogeneity is accounted for to assess herd immunity. The analysis focuses on four cases, where the population is either homogeneous, or is categorized by age cohorts but not by activity levels, or is categorized by different activity levels but not by age, or is categorized both by age and activity levels. Here, we introduce vaccination in a modified version of the case with age cohorts.

## The set-up

### The pandemic model

We employ a modified SIR-model, where there are three groups of individuals ( *A*, *B* and *C*) with different characteristics. In each group $X\in \{A,B,C\}$ there are six categories of individuals:

(i) Susceptible persons ($S_{X}$) who have never been exposed to the virus;
(ii) Infectious persons ($I_{X}$);
(iii) Recovered persons who are no longer infectious and have developed resistance to the virus—these can be divided into two sub-categories:
  - Those who have just ceased to be infectious and still need to (or are required to) stay at home and therefore cannot work ($r_{X}$); and
  - Those who are fully recovered and can work again ($R_{X}$);
(iv) Deceased persons ($D_{X}$);
(v) Vaccinated persons who are immune ($V_{X}^{im}$); and
(vi) Vaccinated persons who are still susceptible ($V_{X}^{s}$).

The dynamics in a SIR-model depend on the recovery rate and the transmission rate. We use a uniform recovery rate $\gamma$ across all groups. Assuming that people are, on average, infectious for five days, implies that $\gamma =0.2$. That is, $\gamma$ measures the rate of transition from $I_{X}$ to $r_{X}$. We assume that people are, on average, absent from work for 15 days, which implies that people remain at home for a further ten days after having ceased to be infectious. We therefore introduce the uniform transition rate $\tau =0.1$ of moving from $r_{X}$ to $R_{X}$. Epidemiologically the distinction between $r_{X}$ and $R_{X}$ has no effect, but in the context of this study it is important to make, because our focus is also on economic outcomes.

In standard pandemic models the transmission rate $\beta$ is homogenous across the entire population, i.e. the rate at which a susceptible individual becomes infected by infectious individuals is $\beta I$, where *I* is the total number (or share) of infectious persons. Here, we instead assume that the rate of transmission varies across different segments of the population, which has been analyzed e.g. in Britton et al. (2020). We employ a simple modification of this approach. Groups *A*, *B* and *C* correspond to age cohorts consisting of young persons (below 20 years of age), middle-aged persons (20 to 59 years of age) and old persons (above 60 years of age), respectively. The shares of these three groups are 0.25, 0.5 and 0.25, roughly corresponding to Swedish population data.

#### Contact patterns

To assess the evolution of the pandemic it is crucial to capture differences in social contact patterns within the population. We use the results from two different sources that have collected data on interactions between different age groups—Wallinga et al. (2006) and Verelst et al. (2021).

Wallinga et al. (2006) uses age-specific Dutch data on face-to-face conversations as a proxy for exposure to infectious respiratory-spread agents. They obtain normalized age-specific contact rates for six cohorts (1–5, 6–12, 13–19, 20–39, 40–59 and 60-). We use the same data, but reduce the number of cohorts to three (1–19, 20–59 and 60-), to obtain the following transmission rates $\beta _{XY}$ between an infected individual in group *Y* and a susceptible person in group *X*, given a general transmission rate of $\beta =0.25$ across the entire population.^7^

**Table:** 

$\beta _{XY}$	1-19 (*A*)	20-59 (*B*)	60- (*C*)
1-19 (*A*)	0.5184	0.1907	0.0690
20-59 (*B*)	0.1907	0.3371	0.1510
60- (*C*)	0.0690	0.1510	0.2945

The values for the general transmission rate and the recovery rate imply that the reproduction rate is 1.25, which was roughly in line with the estimated levels for Sweden by the Public Health Agency of Sweden by the end of 2020.^8^ This relatively low reproduction number is a result of the restrictions on public life that were implemented.

We have also made use of contact data from the Socrates CoMix project intended to measure social distancing during the Covid-19 pandemic. This allows us to shed light on how outcomes from different vaccination strategies could be affected by different policies aimed at restricting contacts. As there are no data from Sweden, we use data collected in Denmark between December 22nd, 2020 and April 16th, 2021 (reported in Verelst et al., 2021). Our analysis thus also uses data collected during a phase in the pandemic when restrictions particularly aimed at protecting the elderly were in place—it should be noted that the time horizon for these measures is likely to be shorter than the duration of a vaccination campaign (except for very high vaccination rates).

Using the same approach as above, we obtain the following alternative transmission rates $\beta _{XY}$ between an infected individual in group *Y* and a susceptible person in group *X*, given a general transmission rate of $\beta =0.25$ across the entire population.^9^

**Table:** 

$\beta _{XY}$	1-19 (*A*)	20-59 (*B*)	60- (*C*)
1-19 (*A*)	0,7124	0,2736	0,0888
20-59 (*B*)	0,2736	0,2682	0,1592
60- (*C*)	0,0888	0,1592	0,2318

Compared to the contact rates obtained in Wallinga et al. (2006), the Danish data feature substantially higher transmission rates among the youngest, while transmissions within the middle-aged group and within the oldest group are fewer. Hence, the Danish contact patterns are more asymmetric, probably reflecting the fact that fewer precautionary measures were targetted at the youngest cohort.

There are several issues that have to be addressed when using contact data collected during the pandemic. For example, the data do not reveal all precautionary measures that have been undertaken, i.e. data reflect quantitative (i.e. number of contacts), but not qualitative (e.g. distancing) impacts of recommendations and policies. Moreover, as vaccination strategies are implemented over a long time horizon it is problematic to use contact data collected during a particular phase of the pandemic.^10^ Nevertheless, it is of interest to analyze how changes in contact patterns due to policy measures might affect outcomes under different strategies.

#### Fatality rates

Data on deaths due to Covid-19 clearly reveal a fatality rate that increases sharply with age. A meta-analysis by Levin et al. (2020) provides estimates of infection fatality rates (i.e. the likelihood of dying from Covid-19 among those infected by the virus) for different cohorts. On the basis of these estimates and Swedish population data for 2019, provided by Statistics Sweden, we obtain the following probabilities of dying per day of being infected: $\delta _{A}=0.00000396017$, $\delta _{B}=0.000268962$ and $\delta _{C}=0.010573046$.

#### The vaccination program

A vaccination program is introduced, such that susceptible persons are vaccinated at rate *u*, which measures the share of the population that gets vaccinated every day. The vaccination rate is $u_{A}$, $u_{B}$ and $u_{C}=u-u_{A}-u_{B}$ for groups *A*, *B* and *C*, respectively; while *u* is assumed to be constant over time (as long as there are still susceptible persons in the population), $u_{A}$, $u_{B}$ and $u_{C}$ change over time in accordance with the chosen vaccination strategy. For example, vaccination strategy *ABC* implies that $u_{A}=u$ and $u_{B}=u_{C}=0$ until $S_{A}=0$ (i.e. until all susceptible group *A* individuals have been vaccinated), whereafter $u_{B}=u$ and $u_{A}=u_{C}=0$ until $S_{B}=0$, followed by $u_{C}=u$ and $u_{A}=u_{B}=0$ until $S_{C}=0$ and the vaccination campaign ends.

The success of a vaccine crucially depends on its efficacy. Efficacy can be measured across several dimensions, e.g. in terms of reducing the likelihood of becoming infected, reducing the probability of becoming hospitalized and reducing the fatality rate. It could be argued that the efficacy is hard to measure as a lower hospitalization rate will increase the chances of survival of those in need of intensive care. Due to a lack of assessments of vaccine efficacy across different dimensions, we simplify the analysis by assuming that vaccine efficacy only relates to transmissibility, without affecting the likelihood of falling ill or dying if being infected.

#### Pandemic dynamics

Given a vaccination rate $u_{X}$ of susceptibles in group *X* and a vaccine efficacy $e\in (0,1]$ the share of immune vaccinated individuals increases by $eu_{X}$ and the share of vaccinated individuals that remain susceptible increases by $(1-e)u_{X}$ in group *X* per day.^11^ For each group *X* the dynamics of the pandemic can be described as follows:

$$\begin{aligned} \overset{.}{S_{X}}&=-(\beta _{XA}I_{A}+\beta _{XB}I_{B}+\beta _{XC}I_{C})S_{X}-u_{X}, \\ \overset{.}{I_{X}}&=(\beta _{XA}I_{A}+\beta _{XB}I_{B}+\beta _{XC}I_{C})S_{X}+(\beta _{XA}I_{A}+\beta _{XB}I_{B}+\beta _{XC}I_{C})V_{X}^{s}-\gamma I_{X}-\delta _{X}I_{X}, \\ \overset{.}{r_{X}}&=\gamma I_{X}-\tau r_{X}, \\ \overset{.}{R_{X}}&=\tau r_{X}, \\ \overset{.}{D_{X}}&=\delta _{X}I_{X}, \\ \overset{.}{V_{X}^{im}}&=eu_{X}, \\ \overset{.}{V_{X}^{s}}&=(1-e)u_{X}-(\beta _{XA}I_{A}+\beta _{XB}I_{B}+\beta _{XC}I_{C})V_{X}^{s}. \end{aligned}$$

For simplicity it will be assumed that $S_{A}$, $I_{A}$, $r_{A}$, $R_{A}$, $D_{A}$, $V_{A}^{im}$, $V_{A}^{s}$, $S_{B}$, $I_{B}$, $r_{B}$, $R_{B}$, $D_{B}$, $V_{B}^{im}$, $V_{B}^{s}$, $S_{C}$, $I_{C}$, $r_{C}$, $R_{C}$, $D_{C}$, $V_{C}^{im}$ and $V_{C}^{s}$ represent shares of the population, i.e. $\sum _{X\in \{A,B,C \}}[S_{X}(t)+I_{X}(t)+r_{X}(t)+R_{X}(t)+D_{X}(t)+V_{X}^{im}(t)+V_{X}^{s}(t)]=1$ at any point in time *t*, where day 1 is the first day that people start being vaccinated. We assume that at day 0, the number of deceased individuals is zero, i.e. $D_{A}(0)=D_{B}(0)=D_{C}(0)=0$; in terms of how vaccination strategies affect outcomes the number of those who have already died from Covid-19 is of no importance. Rather, our focus is on how many more fatalities there will be under different vaccination schemes.

As mentioned above, it is difficult to assess exactly how many people had already been infected when vaccinations started, as there had been many asymptomatic cases or cases with very light symptoms, where it was never established whether these were due to the Corona virus or not. In our calibration we assume that an equal share of 0.1 in all groups belong to the category of recovered people, one-tenth of which are people who cannot yet work, implying that $r_{A}(0)=0.0025$, $R_{A}(0)=0.0225$, $r_{B}(0)=0.005$ $R_{B}(0)=0.045$, $r_{C}(0)=0.0025$ and $R_{C}(0)=0.0225$. Data on new infections per day suggest that by the end of December 2020 (when the first doses of vaccine were administered in Sweden and many other countries) about 0.3 per cent of the Swedish population was infectious. However, although testing capacity had increased considerably at that time, there might still have been many undiscovered Covid-19 cases. We therefore assume that a share of 0.005 in all groups are infected on day 0, i.e. $I_{A}(0)=0.00125$, $I_{B}(0)=0.0025$ and $I_{C}(0)=0.00125$. Hence, the share of susceptibles is 0.895 in all groups, such that $S_{A}(0)=0.22375$, $S_{B}(0)=0.4475$ and $S_{C}(0)=0.22375$.

While we allow for the possibility of vaccinated persons not being immune (whenever the efficacy of the vaccine is below one), we do not account for recovered persons becoming infected again (by the virus variant against which people are vaccinated). In light of reports of people having become infected more than once, this may be a strong assumption. However, the number of persons having been infected by the Covid-19 virus twice was very low at the end of 2020, suggesting that recovery provides immunity at least in the short run.

### Economic environment

Our economy has a single primary factor of production, labor denoted by *L*. This factor is used in manufacturing, the M-sector, and in services, the A-sector. The M-sector is characterized by increasing returns and monopolistic competition. M-sector firms face constant marginal production costs and fixed costs. The A-sector produces a homogeneous good under constant returns to scale, and there is perfect competition in the market for this good. Consumers spend a fixed share of their income on goods from each sector, and have a CES-subutility function dictating their preferences over the various differentiated varieties within the M-sector. All individuals have the utility function

1 $$\begin{aligned} U=C_{A}^{1-\mu }C_{M}^{\mu },\qquad C_{M}=\left[ \int \limits _{0}^{n}c_{i}^{ \frac{\sigma -1}{\sigma }}di\right] ^{\frac{\sigma }{\sigma -1}}, \end{aligned}$$

where $C_{M}$ is consumption of a CES-aggregate of differentiated good, $C_{A}$ is consumption of the A-good, $\mu$ is the consumption share of the M-sector, *n* is the number (mass) of varieties, $c_{i}$ is the amount of variety *i* consumed by an individual and $\sigma >1$ is the elasticity of substitution between varieties. Aggregate income *Y* consists of wage income, i.e. $Y=wL,$ where *w* is the nominal wage rate.

The unit factor requirement of the homogeneous A-sector good is one unit of labor by choice of units. Since the A-good is also chosen as the numeraire,

2 $$\begin{aligned} p_{A}=w=1 \end{aligned}$$

Through utility maximization we obtain demand for variety *i*, which is given by

3 $$\begin{aligned} x_{i}=\frac{p_{i} ^{-\sigma }}{P^{1-\sigma }}L, \end{aligned}$$

where

4 $$\begin{aligned} P\equiv \left( \int \limits _{0}^{n}p_{i}^{1-\sigma }di\right) ^{\frac{1}{ 1-\sigma }} \end{aligned}$$

is the price index of manufacturing goods.

Since firms are identical we drop the firm subscript *i* from now on. Each firm maximizes

5 $$\begin{aligned} \pi =(p-\varphi )x-F, \end{aligned}$$

where $\varphi$ is the unit labour input coefficient and *F* is a fixed production cost. Profit maximization yields the producer price

6 $$\begin{aligned} p^{*}=\frac{\sigma }{\sigma -1}\varphi , \end{aligned}$$

implying that

$$\begin{aligned} P=n^{\frac{1}{1-\sigma }}\frac{\sigma }{\sigma -1}\varphi . \end{aligned}$$

Hence, every firm produces the quantity

$$\begin{aligned} x=\frac{L}{n}\frac{\sigma -1}{\sigma \varphi }, \end{aligned}$$

which implies that its profits are given by

7 $$\begin{aligned} \pi =\frac{L}{\sigma n}-F \end{aligned}$$

It follows then from the zero-profit condition that the equilibrium number of firms is given by

8 $$\begin{aligned} n^{*}=\frac{L}{\sigma F}. \end{aligned}$$

Thus, the produced quantity is given by

$$\begin{aligned} x^{*}=\frac{\sigma -1}{\varphi }F. \end{aligned}$$

The indirect utility in equilibrium is therefore given by

9 $$\begin{aligned} V^{*}=\frac{wL}{P^{*\mu }p_{A}^{1-\mu }}=\left( n^{*\frac{1}{ 1-\sigma }}\frac{\sigma }{\sigma -1}\varphi \right) ^{-\mu }L=L^{\frac{ \sigma -1+\mu }{\sigma -1}}\left( \frac{\sigma -1}{\varphi }\right) ^{\mu }\left( \sigma F\right) ^{\frac{\mu }{1-\sigma }}. \end{aligned}$$

This equation gives a measure of the real GDP, that we will use in our simulations of the economic consequences of different vaccination strategies.

### Parametrization

While many infectees only suffer light symptoms and may still be able to work from home we make the simplifying assumption that productivity is zero for all persons belonging to categories $I_{X}$ or $r_{X}$. For non-infected (and non-deceased) individuals it is assumed that productivity is 0 per group *A* individual and day, while it is 0.1 per group *C* individual and day. Hence, no young person contributes to output, while old people make a contribution roughly corresponding to the number of 60–64 year olds in relation to the number of group *B* individuals.

However, working hours of non-infected persons are also negatively affected by increasing numbers of infectees, e.g. through quarantines and restrictions. We therefore assume that working hours by non-infected persons are reduced by a factor. More specifically, this factor is given by $1-\left[ I_{A}(t)+I_{B}(t)+I_{C}(t)\right] ^{\lambda }$. Thus, the normalized total labour force at any day *t* is given by

$$\begin{aligned} L(t)=\left\{ 1-\left[ I_{A}(t)+I_{B}(t)+I_{C}(t)\right] ^{\lambda }\right\} \left\{ \begin{array}{c} S_{B}(t)+R_{B}(t)+V_{B}^{im}(t)+V_{B}^{s}(t) \\ +0.1\left[ S_{C}(t)+R_{C}(t)+V_{C}^{im}(t)+V_{C}^{s}(t)\right] \end{array} \right\} . \end{aligned}$$

Using Swedish working hours data for 2020 we set $\lambda =0.46$.^12^ To assess the economic consequences of the pandemic we use a fairly generic $\sigma =5$ in Eq. (9) to obtain a measure for real GDP.^13^ As a benchmark we use the outcome in the absence of a vaccine, such that we are able to calculate the economic gain during one year in relation to the vaccination rate.

## Simulations

To evaluate the implications of different vaccination strategies we will focus on the following measures:

(i) The share of the population that will have deceased two years after vaccinations have started.
(ii) The share of the population that will have been infected two years after vaccinations have started.
(iii) The peak of the share of infectious individuals.
(iv) Economic gains from vaccinations one year after their start, measured as the percentage gain in output in relation to one year’s output in the absence of a vaccine.

We simulate the outcomes for six different vaccination strategies (*ABC*, *ACB*, *BAC*, *BCA*, *CAB*, and *CBA*), according to which susceptible individuals in the three age groups are vaccinated in sequence.^14^ The daily vaccination rate *u* measures the share of the entire population that gets vaccinated every day, reflecting the capacity for vaccinations. In our analysis we let *u* take on values between 0 and 0.01. Hence, the upper bound implies that 1 per cent of the entire population is vaccinated every day, which would be hard to implement in most countries. Note that a vaccination program where 1 per cent of the population gets vaccinated every day will lead to all susceptible individuals being covered in less than one hundred days as some individuals will get infected and possibly also die before being offered the vaccine Moreover, different vaccination strategies will impact on the pandemic dynamics differently, such that the time it take to cover all susceptible individuals will also differ for a given *u*.

In our simulations we assume a general transmission rate of $\beta =0.25$ and a vaccine efficacy of $e=0.9$. The sensitivity of our results with regard to changes in these parameter values is examined in Sect. Sensitivity analysis. Although we have calibrated the transmission rates in the Dutch and the Danish data to the same general transmission rate of 1.25, the evolution of the pandemic will differ as transmission rate patterns are different. Table 1 shows the expected outcomes under the two alternative contact patterns in the absence of any vaccine.

**Table 1:** Outcomes in the absence of vaccinations given different contact patterns

	Wallinga et al. (2006)	CoMix Denmark 2021
Fatalities	1294 per million	2121 per million
Total share of infected	23.9 %	35.4 %
Peak share of infectees	0.587 %	1.516 %

Clearly the Danish contact patterns would yield worse outcomes. The main reason is that the significantly higher transmission rates among the youngest trigger a faster spread of the disease in the entire population, leading to higher numbers in terms of the peak share of infectees, the total share of infected and fatalities. In what follows we present the outcomes of the different vaccination strategies when using the transmission rates based on the Wallinga et al. (2006) and the CoMix Denmark contact patterns, respectively.

### Fatalities

**Fig. 1:**
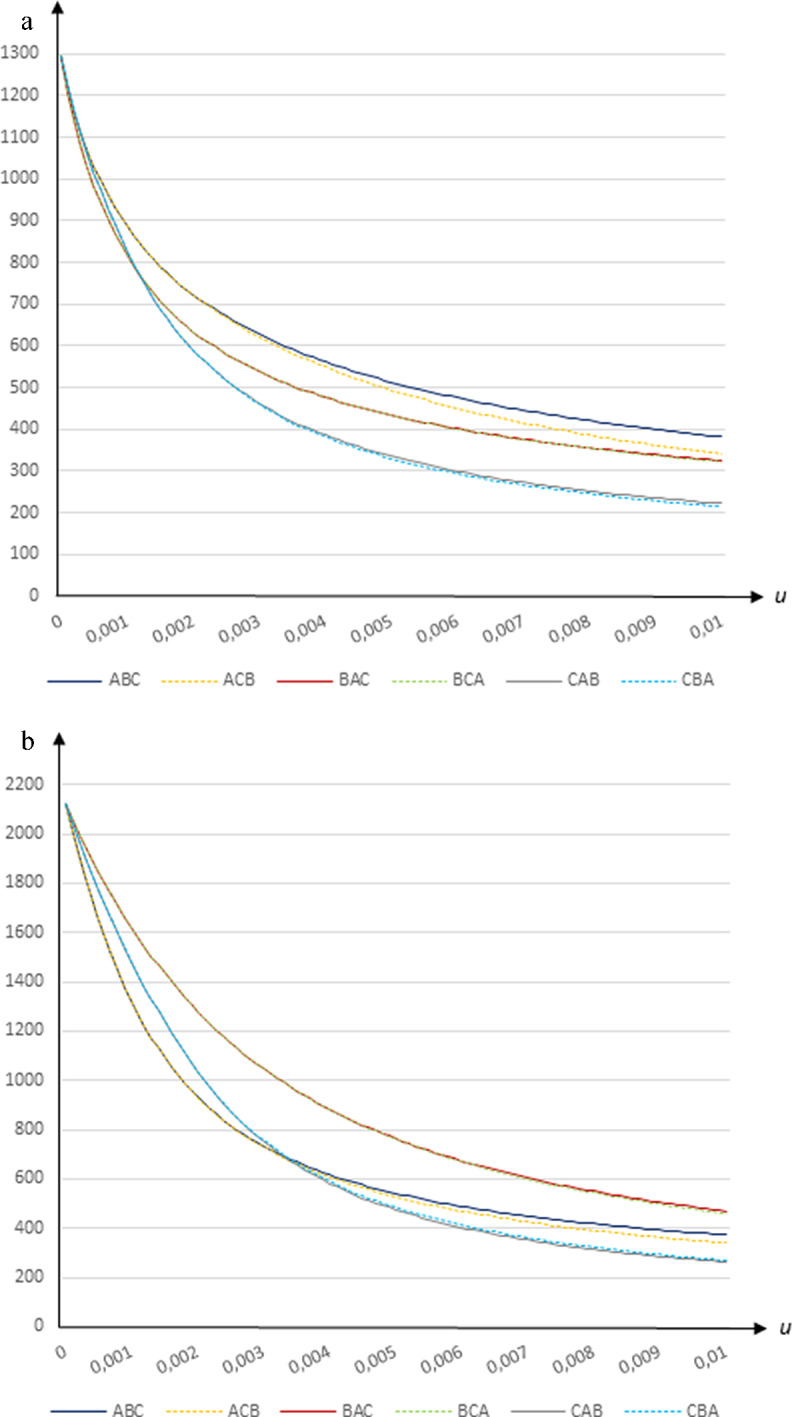
The number of deceased (per million) in relation to the daily vaccination rate *u* when *β* = 0.25 and *e* = 0.9 using **a** the Wallinga et al. (2006) contact patterns and **b** the CoMix Denmark contact patterns

The total number of fatalities decreases sharply in the vaccination rate. Using transmission rates based on Wallinga et al. (2006), the rate drops from 1294 deaths per million in the absence of a vaccination program to 215–382 fatalities per million for $u=0.01$ (such that it would take 86–88 days to cover all susceptible individuals) as shown in Fig. 1a, and vaccinating group *A* (the young) first leads to most fatalities for all vaccination rates. Using transmission rates based on CoMix Denmark data, the rate falls from 2121 deaths per million in the absence of a vaccination program to 262-470 fatalities per million for $u=0.01$ (such that it would take 83–87 days to cover all susceptible individuals) as shown in Fig. 1b. In this case vaccinating group *B* (the middle-aged) first leads to most fatalities for all vaccination rates.

A general conclusion is that rapidly creating vaccination capacities is crucial to keep the number of deceased as low as possible. However, our simulations also reveal significant differences between the strategies under consideration. Which strategy minimizes fatalities crucially depends on the vaccination rate. Vaccinating the elderly (group *C*) first minimizes the total number of deaths given that the vaccination rate is sufficiently high, above $u=0.0012$ when the Wallinga et al. (2006) contact patterns are used and above $u=0.0033$ when the CoMix Denmark contact patterns are used. However, first vaccinating group *B* (the middle-aged) minimizes fatalities for vaccination rates below 0.0012 (implying that it would take 684 days to cover the entire susceptible population under strategies *BAC* and *BCA*) with transmission rates based on Wallinga et al. (2006). In contrast, when contact patterns based on CoMix Denmark are used, first vaccinating group *A* (the young) minimizes fatalities for vaccination rates below 0.0033 (implying that it would take 252 days to cover the entire susceptible population under strategies *ABC* and *ACB*).

Hence, vaccinating the oldest group first minimizes fatalities for sufficiently high vaccination rates. But when vaccination proceeds slowly, it is more important to dampen the spread of the virus to protect the elderly. Under these conditions the total number of deaths are minimized by first vaccinating the group that drives transmission in the population - the middle aged group with the Wallinga et al. (2006) contact patterns (it is the largest group and is crucial for transmissions across age groups) and the young group with the CoMix Denmark contact patterns (its transmission rates are much higher).

It is of little importance which group gets vaccinated secondly when the vaccination rate is low. But at higher vaccination rates there are differences in outcomes depending on which group gets vaccinated secondly. In particular, there is a substantial and increasing difference in outcomes between strategies *ABC* and *ACB*, with *ABC* yielding a higher number of deaths at all vaccination rates for both contact matrices.

### Economic gains from vaccination

**Fig. 2:**
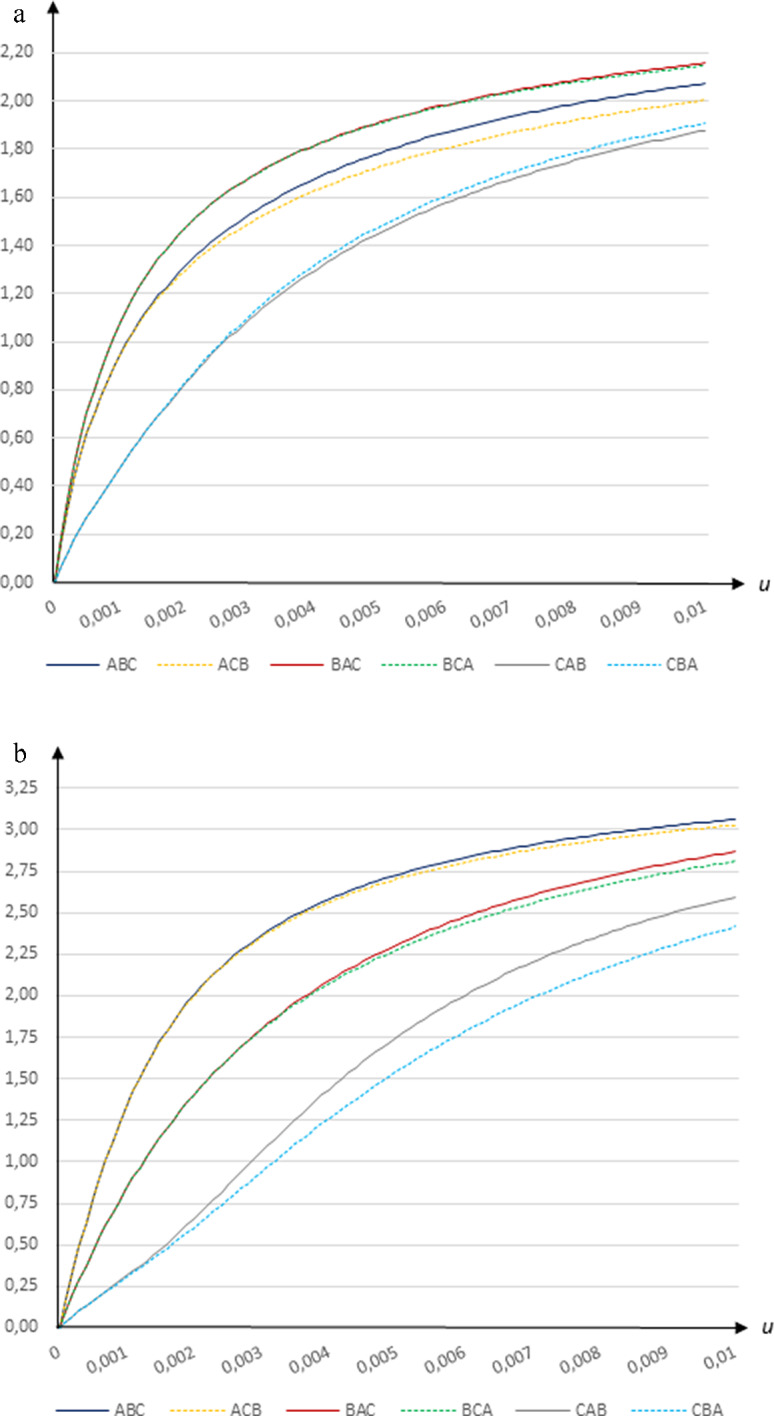
The economic gain from vaccinations (per cent) in relation to the daily vaccination rate *u* when *β* = 0.25 and *e* = 0.9 using **a** the Wallinga et al. (2006) contact patterns and **b** the CoMix ﻿Denmark contact patterns

Economic gains increase in the vaccination rate, reaching 1.88–2.16 per cent of GDP for $u=0.01$ with transmission rates based on Wallinga et al. (2006), as shown in Fig. 2a, and reaching 2.42–3.96 per cent of GDP for $u=0.01$ with transmission rates based on CoMix Denmark, as shown in Fig. 2b.

Our simulations suggest that there are substantial economic gains from increasing the vaccination rate. For instance, using transmission rates based on Wallinga et al. (2006), an acceleration of the vaccination rate from $u=0.0025$ (lasting 324 days to cover the entire susceptible population) to $u=0.005$ (lasting 168 days) under the fatality-minimizing strategy *CBA* would lead to a gain of roughly 0.5 per cent of GDP (2.3 billion USD/25 billion SEK for Sweden).^15^ Moreover, this increase in the vaccination rate would lead to a decrease in fatalities of 186 per million (1932 fewer deaths in Sweden) and a decrease of 2.51 per cent in the total share of infected people (261 040 fewer cases in Sweden), while also slightly reducing the peak infection rate. If instead the CoMix Denmark contact patterns are used, the same increase in the vaccination rate from $u=0.0025$ (lasting 284 days to cover the entire susceptible population) to $u=0.005$ (lasting 155 days) under the fatality-minimizing strategy *CAB* would lead to a gain of roughly 0.9 per cent of GDP (4.2 billion USD/45 billion SEK for Sweden), while decreasing fatalities by 400 per million (4160 fewer deaths in Sweden), reducing the total share of infected people by 6.61 per cent (687 440 fewer cases in Sweden) and decreasing the peak infection rate by 0.17 per cent.

Using contact patterns based on Wallinga et al. (2006), vaccinating group *B* first yields the highest economic gains, while vaccinating group *C* first leads to the smallest gains from vaccinations. Hence, for vaccination rates above 0.0012 such that vaccinating the elderly first minimizes the total number of deaths there is a trade-off between fatalities and economic outcomes. For example, given a vaccination rate of $u=0.0025$ (lasting 324 days), the economic gain generated by switching from the fatality-minimizing strategy *CBA* to vaccinating group *B* first is about 0.61 per cent of GDP (2.8 billion USD/30.5 billion SEK for Sweden). In addition, such a switch would imply a reduction in the total share of infected people of 3.13 per cent (about 325 000 fewer cases in Sweden); however, it would also lead to an increase in fatalities by 64 per million (668 cases in Sweden) as more old persons would become exposed to the virus.

Using transmission rates based on CoMix Denmark, vaccinating the elderly first leads to the lowest number of deaths at vaccination rates above 0.0033. Since vaccinating the youngest first yields the highest economic gains at all vaccination rates a trade-off emerges at higher vaccination rates. For instance, at a vaccination rate of $u=0.0075$ (lasting 108 days), the economic gain generated by switching from the fatality-minimizing strategy *CAB* to the economically most optimal strategy *ABC* is about 0.65 per cent of GDP (3 billion USD/32.5 billion SEK for Sweden). While this switch would also lead to a reduction in the total share of infected people of 4.67 per cent (about 485 680 fewer cases in Sweden), it would imply an increase in fatalities by 101 per million (1050 cases in Sweden).

### Total share of infected

**Fig. 3:**
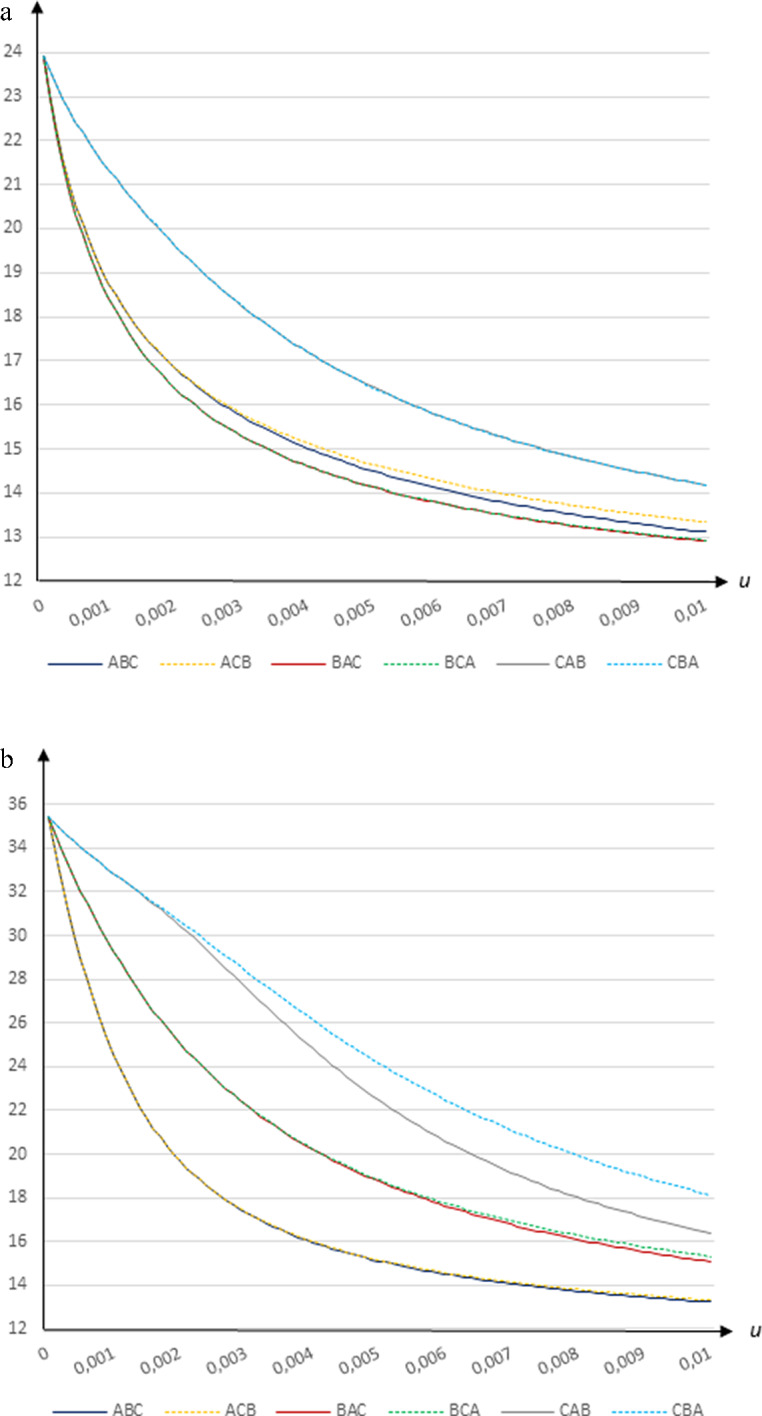
The total share of infected people (per cent of population) in relation to the daily vaccination rate *u* when *β* = 0.25 and *e* = 0.9 using **a** the Wallinga et al. (2006) contact patterns and **b** the CoMix ﻿Denmark contact patterns

The share of the population that will have been infected by the virus decreases substantially in the vaccination rate. Using transmission rates based on Wallinga et al. (2006) the decline is from 23.9 per cent for $u=0$ to 12.9-14.2 per cent for $u=0.01$, as shown in Fig. 3a. Using the transmission rates based on CoMix Denmark the share of infected persons falls from 35.4 per cent for $u=0$ to 13.2-18.1 per cent for $u=0.01$, as illustrated by Fig. 3b.

It turns out that vaccinating group *C* first clearly yields the highest total number of infected people in both cases. Using transmission rates based on Wallinga et al. (2006), vaccinating group *B* first leads to the lowest total number of infected persons at all vaccination rates, while vaccinating group *A* first implies the lowest total number of infected people when transmission rates based on CoMix Denmark are used.

The share of the population being infected is thus minimized by first vaccinating the group that drives the pandemic. The difference between vaccinating this group first and vaccinating the elderly first is largest at intermediate vaccination rates. For example, using contact patterns based on Wallinga et al. (2006) and given a vaccination rate of 0.0025 (such that it would take 325–337 days to vaccinate the entire population), 18.9 per cent of the population will have been infected under strategy *CBA*, whereas 15.8 per cent of the population will have been infected under strategy *BAC*. If instead transmission rates based on CoMix Denmark are used, a vaccination rate of 0.0025 (such that it would take 282-326 days to vaccinate the entire population) leads to 29.5 per cent of the population becoming infected under strategy *CBA*, whereas 18.3 per cent of the population will have been infected under strategies *ABC* and *ACB*.

### Peak share of infectees

**Fig. 4:**
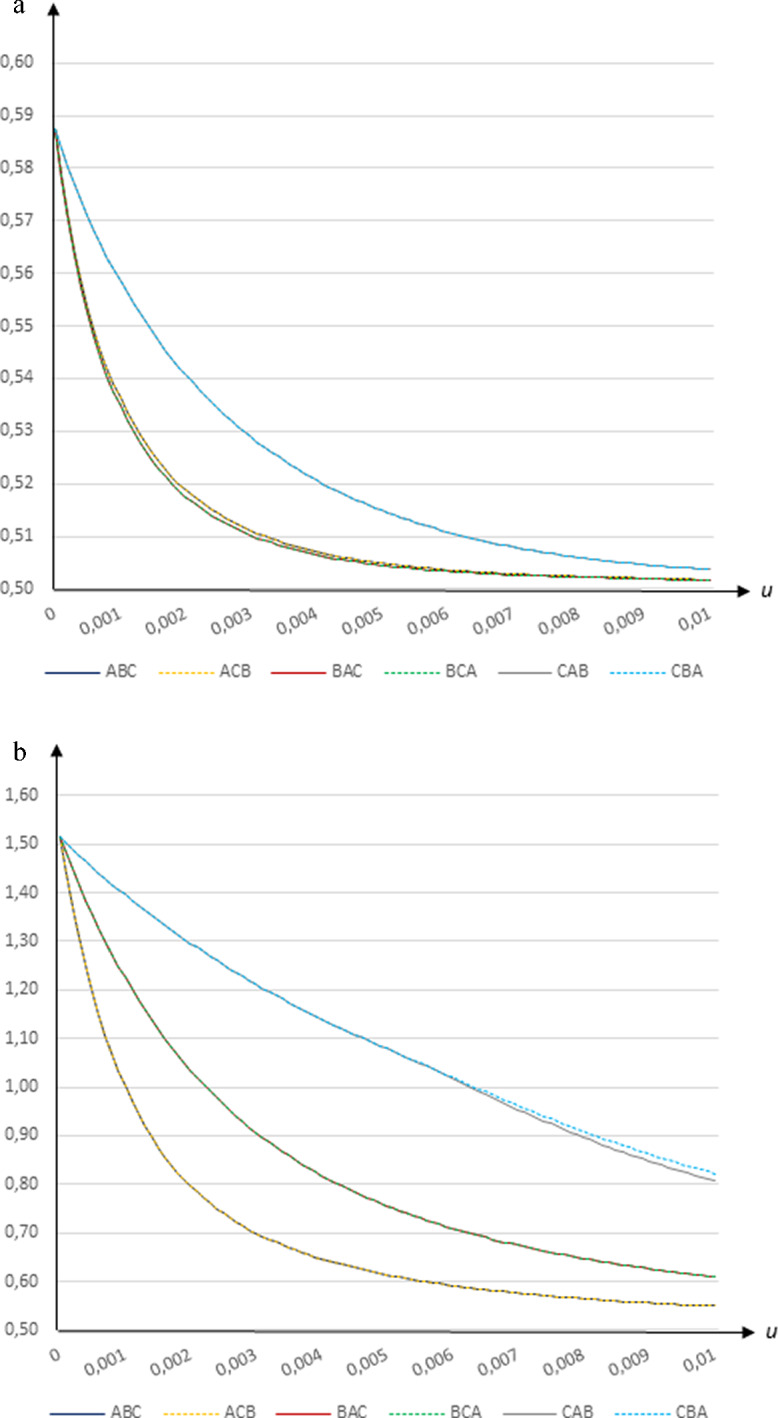
The peak share of infected people (per cent of population) in relation to the daily vaccination rate *u* when *β* = 0.25 and *e* = 0.9 using **a** the Wallinga et al. (2006) contact patterns and **b** the CoMix ﻿Denmark contact patterns﻿

The vaccination program is implemented in the midst of the pandemic (the total share of infected people is 0.5 per cent on day 0) and the transmission rates are relatively low due to the imposed restrictions (the general transmission rate is $\beta =0.25$ across the entire population). Figure 4a and b illustrate how the peak share of infectees is reduced as the vaccination rate increases under different strategies.

Assuming contact patterns based on Wallinga et al. (2006), the infection rate does not increase dramatically even in the absence of vaccinations, and the infection rate peaks relatively early (on day 38 for $u=0$ and in less than a week for $u=0.01$). The peak share of infectees decreases in the vaccination rate, from 0.587 per cent for $u=0$ to 0.502–0.504 per cent for $u=0.01$. Vaccinating group *B* first yields the best outcome, but the difference to vaccinating group *A* first is very small. In contrast, vaccinating group *C* first leads to a somewhat higher peak infection rate, especially at intermediate vaccination rates, but the difference compared to when group *B* gets vaccinated first is never higher than 0.0245 per cent, which implies a difference of about 2500 cases in Sweden.^16^

Using transmission rates based on CoMix Denmark, the peak infection rate increases substanstially at low vaccination rates (to 1.52 per cent on day 54 for $u=0$). As the vaccination rate increases the peak rate falls to between 0.549 on day 8 when group *A* is vaccinated first and 0.822 on day 29 under strategy *CBA* for $u=0.01$. By vaccinating the youngest group first the pandemic is contained most quickly, thus yielding substantially lower peak infection rates. At intermediate vaccination rates the difference in peak infection rate between vaccinating group *A* first and vaccinating group *C* first is substantial, reaching a maximum of 0.5131 per cent, implying a difference of 53 362 cases in Sweden.

### Summary

The simulations where $\beta =0.25$ and $e=0.9$ provide clear-cut results for both contact patterns. What matters most to outcomes is which group gets vaccinated first, while it is much less important which group gets vaccinated next.

If the main policy objective is to reduce fatalities, it is optimal to start vaccinating group *C* for sufficiently high vaccination rates, such that it takes less than 684 days to cover the entire susceptible population when transmission rates based on Wallinga et al. (2006) are used and less than about 252 days to cover the entire population when transmission rates based on CoMix Denmark are used.

If capacities for swift vaccinations of the population are limited, it is unambiguously optimal to start vaccinating the group that drives the pandemic first. Thus, it is optimal to vaccinate group *B* first if the vaccination rate is so low that covering the entire population takes more than 684 days given contact patterns based on Wallinga et al. (2006), while it is optimal to vaccinate group *A* first when covering the entire population takes more than 252 daýs given contact patterns based on CoMix Denmark. Targetting the group that drives the pandemic will in this case not only minimize fatalities, but also lead to the lowest total number of infected persons, the lowest peak share of infectees and the largest economic gains.

In contrast, we obtain a trade-off between minimizing fatalities and the other three outcome variables at higher vaccination rates. Vaccinating group *C* implies less deaths, but it comes at the cost of a higher share of the population becoming infected and therefore also a smaller economic gain from vaccinations. The negative consequences of vaccinating group *C* rather than the group that drives the pandemic first are largest at intermediate vaccination rates.

### The trade-off between reducing fatalities and achieving better economic outcomes

**Fig. 5:**
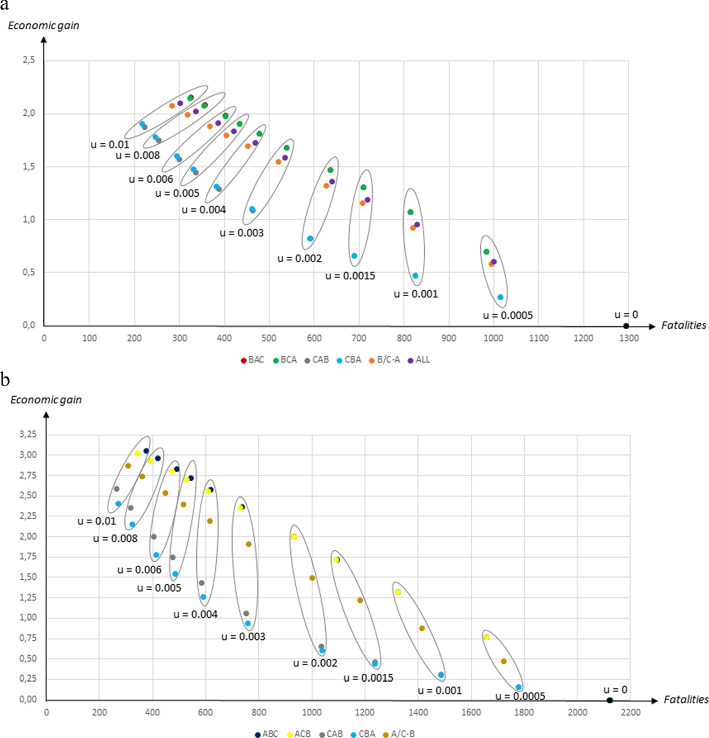
Economic gains (per cent) in relation to fatalities (per million people) for different vaccination strategies and different daily vaccination rates *u* using **a** the Wallinga et al. (2006) contact patterns and **b** the CoMix ﻿Denmark contact patterns﻿

Figure 5a and b illustrate the trade-off between reducing fatalities and enhancing economic growth that emerges for sufficiently high vaccination rates. In Fig. 5a, which is based on the Wallinga et al. (2006) contact patterns, strategies *ABC* and *ACB* are not included as they yield worse outcomes than strategies *BAC* and *BCA* both in terms of fatalities and economic gains. Likewise, strategies *BAC* and *BCA* are not included in Fig. 5b, which is based on the CoMix Denmark contact patterns, as strategies *ABC* and *ACB* lead to better outcomes both in terms of fatalities and economic gains.

We have also included two strategies that have not been considered hitherto in Fig. 5a: strategy *B*/*C*-*A*, under which first age groups *B* and *C* are vaccinated simultaneously before the youngest group gets vaccinated; and strategy *ALL*, under which the entire population is vaccinated simultaneously. These strategies are not considered in Fig. 5b, because they yield worse outcomes than strategy *ACB* both in terms of fatalities and economic gains when the CoMix Denmark contact patterns are used; instead strategy *A*/*C*-*B*, under which first age groups *A* and *C* are vaccinated simultaneously before the middle-aged group gets vaccinated, is also included.

*The* Wallinga et al. (2006) *contact pattern*

It is easy to see that *BAC* and *BCA* yield better outcomes both in terms of fatalities and economic growth compared to the other strategies for low vaccination rates; outcomes under *BAC* and *BCA* are almost identical. At higher vaccination rates *CBA* yields unambiguously better outcomes than *CAB*, while a trade-off emerges between strategies *CBA*, *B*/*C*-*A*, *ALL*, *BCA* and *BAC*. It is not obvious which would be chosen by a social planner who has to weigh fatalities agains economic gains. It is thus possible that some strategy other than the ones focused upon here yields socially optimal outcomes at high vaccination rates.

*The CoMix Denmark contact pattern*

If the CoMix Denmark contact patterns are used, strategies *ABC* and *ACB* yield better outcomes both in terms of fatalities and economic growth compared to the other strategies for low vaccination rates. At higher vaccination rates *CAB* yields unambiguously better outcomes than *CBA*, while a trade-off emerges between strategies *CAB*, *A*/*C*-*B*, *ABC* and *ACB*. Also in this case some strategy other than the ones focused upon here might yield socially optimal outcomes at high vaccination rates.

## Sensitivity analysis

In what follows we examine how sensitive the above results are to changes in the parameter values for the efficacy of the vaccine and the general transmission rate.

### Vaccine efficacy

Naturally, a higher efficacy of the vaccine is associated with a lower fatality rate, a lower total number of infected people and a lower peak share of infectees, as well as larger economic gains from vaccinations for all strategies under consideration. Table 2 shows how outcomes improve as the efficacy rate increases from $e=0.5$ to $e=1$, given a general transmission rate $\beta =0.25$ and a daily vaccination rate $u=0.0025$.

**Table 2:** Outcomes for different contact patterns and for different vaccine efficacy rates *e* when $\beta =0.25$ and $u=0.0025$

	Wallinga et al. (2006)		CoMix Denmark 2021	
	$e=0.5$	$e=1$	$e=0.5$	$e=1$
Fatalities (per million)	728–800	476–648	1131–1473	767–1092
Total share of infected (%)	17.4–20.2	15.5–18.6	22.2–31.6	17.7–29.1
Peak share of infectees (%)	0.53–0.55	0.51–0.53	0.90-1.35	0.72–1.23
Economic gains (%)	0.73–1.27	1.01–1.64	0.51–1.64	0.83–2.30

The effects of a lower vaccine efficacy resembles in some ways the effect of a lower vaccination rate. Using the Wallinga et al. (2006) contact patterns, the threshold value for *u*, below which first vaccinating the middle aged (group *B*) minimizes fatalities, decreases in the efficacy rate; it is 0.0025 (such that it would take 336 days to vaccinate the entire susceptible population under strategies *BAC* and *BCA*) when $e=0.5$ and 0.0011 (such that it would take more than two years to vaccinate all susceptible persons under all vaccination strategies) when $e=1$.^17^

Using the CoMix Denmark contact patterns, first vaccinating the young (group *A*) minimizes fatilities for all vaccination rates whenever the efficacy rate is below $e=0.64$. As the efficacy rate increases above $e=0.64$ the threshold value for *u*, below which first vaccinating the youngest (group *A*) minimizes fatalities, decreases to 0.0027 (such that it would take 304 days to vaccinate the entire susceptible population under strategies *ABC* and *ACB*) for $e=1$.^18^

### Transmission rates

If the transmission rates increase uniformly, this will obviously lead to more fatalities, a higher total share of infected persons and a higher peak number of infectees at all vaccination rates for all strategies. Not surprisingly, the economic gain from vaccinations also increases as transmission rates increase.

As shown in Table 3 we obtain qualitatively similar results regarding which strategies are best in terms of our outcome measures, with one important exception. Using the Wallinga et al. (2006) contact patterns, it is optimal to implement strategy *CBA* to minimize fatalities at all vaccination rates for general transmission rates below 0.205 and above 0.271. Using the CoMix Denmark contact patterns, it is optimal to implement strategy *CAB* to minimize fatalities at all vaccination rates for general transmission rates below 0.157 and above 0.272.

The gains from increasing the vaccination rate in terms of the four outcome measures become more pronounced for higher transmission rates. The following table show how outcomes improve as the vaccination rate increases from $u=0$ to $u=0.01$ and how these outcomes are affected by an increase in the general transmission rate from $\beta =0.25$ to $\beta =0.3$, given a vaccine efficacy of $e=0.9$.

**Table 3:** Outcomes for different contact patterns and for different daily vaccination rates *u* for $\beta =0.25$ and $\beta =0.3$ given that $e=0.9$

	Wallinga et al. (2006)			CoMix Denmark 2021		
	$u=0$	$u=0.005$	$u=0.01$	$u=0$	$u=0.005$	$u=0.01$
$$\beta =0.25$$						
Fatalities (per million)	1294	330–514	215–382	2121	475–764	262–470
Total share of infected (%)	23.9	14.1–16.4	12.9–14.2	35.4	15.1–24.2	13.2–18.1
Peak share of infectees (%)	0.59	0.50–0.51	0.50–0.50	1.52	0.61–1.08	0.55–0.82
Economic gains (%)	0	1.45–1.91	1.88–2.16	0	1.55–2.73	2.42–3.06
$$\beta =0.3$$						
Fatalities (per million)	3383	712–1446	357–826	3908	1038–1987	487–1098
Total share of infected (%)	44.6	20.1–28.5	15.5–19.8	52.9	22.3–40.3	16.1–29.0
Peak share of infectees (%)	2.49	0.98–1.61	0.74–1.12	4.43	1.39–3.40	0.90–2.44
Economic gains (%)	0	1.81–3.05	2.98–3.72	0	1.24–2.89	2.52–3.75

Clearly higher rates of transmission make increasing vaccination capacities more urgent to avoid a high number of deaths. As noted above, group *C* should be vaccinated first at all vaccination rates when the virus spreads quickly to reduce the number of deaths among the elderly. Also in terms of the total number of infected people, increases in the vaccination rate lead to substantially better outcomes. The difference between vaccinating the group that drives the pandemic rather than the elderly first becomes larger when the virus spreads more quickly, in particular at intermediate vaccination rates.

The impact of an increasing vaccination rate on the peak infection rate becomes much stronger when the general transmission rate increases. It also clearly demonstrates that vaccinating the group that drives the pandemic rather than the elderly first yields substantially better outcomes, especially at intermediate vaccination rates, which is a crucial factor to account for to avoid the health care system becoming overwhelmed in case the virus spreads quickly.

In terms of economic gains the results in Sect. Peak share of infectees are confirmed. Vaccinating the group that drives the pandemic first yield the best economic outcomes, but the difference between the economic outcomes of the different strategies is reduced at high vaccination rates.

To summarize, although the general pattern becomes more pronounced, it remains qualitatively the same for higher transmission rates. Fatalities are substantially lower when group *C* gets vaccinated first, while vaccinating the group that drives the pandemic first leads to considerably lower total and peak shares of infectees, as well as higher economic gains from vaccinations. Thus, the trade-off between these two alternative approaches becomes more apparent.

### Quantifying the trade-off between fatalities and the other outcome variables

Under the Wallinga et al. (2006) contact patterns, given a vaccination rate of $u=0.0025$ (implying that it would take 256 days to cover all susceptible persons) and an efficacy of $e=0.9$, the fatality-minimizing strategy *CBA* leads to 1382 deaths per million, 36.1 per cent of the population becoming infected, a peak infection rate of 1.95 per cent and economic gains of 0.92 per cent, while implementing strategy *BAC* (which would last for 293 days) would lead to 1771 deaths per million, 27.0 per cent of the population becoming infected, a peak infection rate of 1.37 per cent and economic gains of 2.14 per cent. In the Swedish case, choosing strategy *CBA* rather than strategy *BAC* would imply 4042 fewer deaths, but almost one million more infected persons, a peak number of infected people about 60 000 higher and foregone economic gains of 1.22 per cent of GDP (5.67 billion USD/61 billion SEK).

Using the CoMix Denmark contact patterns, given a vaccination rate of $u=0.0025$ (implying that it would take 214 days to cover all susceptible persons) and an efficacy of $e=0.9$, the fatality-minimizing strategy *CAB* leads to 1983 deaths per million, 46.6 per cent of the population becoming infected, a peak infection rate of 3.85 per cent and economic gains of 0.56 per cent, while implementing strategy *ABC* (which would last for 272 days) would lead to 2334 deaths per million, 32.0 per cent of the population becoming infected, a peak infection rate of 2.22 per cent and economic gains of 1.77 per cent. In the Swedish case, choosing strategy *CAB* rather than strategy *ABC* would imply 3686 fewer deaths, but 1.5 million more infected persons, a peak number of infected people about 170 000 higher and foregone economic gains of 1.21 per cent of GDP (5.67 billion USD/61 billion SEK).

The gains from accelerating the administration of vaccines are more substantial at higher transmission rates. By doubling the vaccination rate to $u=0.005$ (such that it would take 144 days to vaccinate all susceptible persons) when implementing strategy *CBA*, fatalities would be reduced by 670 per million, the total share of infected persons would decrease by 7.7 per cent, the peak infection rate would be 0.34 per cent lower and economic gains would be 1.01 per cent higher In the Swedish case this would imply almost 7000 fewer deaths, about 800 000 fewer infected persons, a peak number of infected about 35 000 lower and economic gains of 6.06 billion USD (50, 5 billion SEK).

Using the CoMix Denmark contact pattern, by doubling the vaccination rate to $u=0.005$ (such that it would take 123 days to vaccinate all susceptible persons) when implementing strategy *CAB*, fatalities would be reduced by 945 per million, the total share of infected persons would decrease by 7.9 per cent, the peak infection rate would be 0.45 per cent lower and economic gains would be 0.78 per cent higher For Sweden this would lead to almost 10 000 fewer deaths, more than 800 000 fewer infected persons, a peak number of infected about 47 000 lower and economic gains of 3.63 billion USD (39 billion SEK).

## Conclusions

We analyze a vaccine campaign against Covid 19 in a stylized model with three age groups that are roughly calibrated to Swedish demographic data. The age groups differ with respect to their fatality rates. Crucially, we also account for heterogeneity in contact patterns within and between age groups, such that the transmission parameters are specific to each pair of age groups.

A vaccine campaign can either prioritize the most fragile part of the population to protect them from the infection or prioritize to quickly eradicate the infection, in which case age groups with high transmission rates should be vaccinated first. We show that fatalities are minimized by first vaccinating the elderly, whenever the general transmission rate is sufficiently high or the vaccination rate is sufficiently high or the vaccine efficacy is sufficiently high. However, for some combinations of low vaccination rates and low transmission rates (e.g. due to restrictions) deaths are minimized by first vaccinating the group that drives the pandemic; the lower is the efficacy of the vaccine, the wider is the range of vaccinations rates, for which this is true.

A policy implication for countries where vaccinations cannot progress at a high rate and the vaccine efficacy is not so high might therefore be to impose further restrictions in order to protect the elderly and to start vaccinating younger groups first. Thereby deaths would be minimized, while at the same time the spread of the disease would be ccountered most efficiently.

In terms of other outcome measures such as the total number and the peak number of infected persons it is always best to start vaccinating the group that drives the infection first. When it comes to the economic gains from vaccinations it is always best to start vaccinating the group that drives the pandemic first. However, the advantage of starting with this group decreases for high vaccination rates. We also demonstrate that there are very substantial economic gains, in addition to the health benefits, from a speedy vaccination campaign. In our model we obtain a benchmark for the gains from doubling the vaccination rate, such that covering the entire susceptible population would take about 170 rather than about 330 days, of approximately 0.5 per cent of GDP.

## Footnotes

1 See e.g Luyten et al. (2020) and Roope et al. (2020).

2 This type of epidemiological model was introduced by Kermack and McKendrick (1927).

3 An aspect not considered here is that transmission rates also differ among professions. Occupation-based infection risks are estimated by e.g. Babus et al. (2020).

4 See also Gimma et al. (2022), who use CoMix data from surveys conducted in England during nine different phases of the Covid-19 pandemic (between March 2020 and March 2021).

5 We also do not consider new variants emerging during the vaccination campaign. However, by analyzing the effect of a lower vaccine efficacy we implicitly account for the possibility of new variants, against which the vaccine is not as effective.

6 Hodgson et al. (2020) thoroughly discuss the definition of efficacy in relation to potential Covid-19 vaccines. As they point out, "many different endpoints are used in vaccine research to define efficacy depending on the pathogen, consequences of infection, and transmission dynamics." Furthermore, "outcomes might include reduction in infection (i.e., assessing sterilising immunity), severity of resultant clinical disease (i.e., assessing disease-modifying immunity), or duration of infectivity." Here we use the share of vaccinated persons that become immune for vaccine efficacy. Data for this is widely available, whereas, for instance, reducing the serverity of the infection is harder to measure.

7 More specifically, we use the normalized age-specific contact rates (after correction for reciprocity) of Appendix Table 2 in Wallinga et al. (2006) to calculate the total number of reported weekly contacts for every age group (1–5, 6–12, 13–19, 20–39, 40–59, 60-). Next, we use these numbers to calculate the total number of reported weekly contacts for our cohorts (1-19, 20-59, 60-), which we then, following Diekmann et al. (1990), transform into a next-generation transmission matrix that is adjusted so that its largest eigenvalue equals $R_{0}=1.25$, implying a general transmission rate of $\beta =0.25$ among the entire population given that $\gamma =0.2$.

8 https://www.folkhalsomyndigheten.se/smittskydd-beredskap/utbrott/aktuella-utbrott/covid-19/statistik-och-analyser/analys-och-prognoser/

9 In CoMix there are three cohorts (0–17, 18–59 and 60-) that do not exactly match the ones we obtained from the Wallinga et al. (2006) data. To simplify our analysis we assume that the contact data for the young and middle-aged groups represent the same age span as in Wallinga et al. (2006), i.e. we assume that contact patterns for 1–19 year olds and 20–59 year olds correspond to those identified for 0–17 year olds and 18–59 year olds in the CoMix data set.

10 Gimma et al. (2022) present contact data collected during nine different phases of the Covid-19 pandemic in England between March 2020 and March 2021. These data shed light on how different policies in terms of lockdowns and restrictions affected contact patterns within and between different age groups. The data collection phases vary in length, between two weeks and more than ten weeks.

11 We thus assume that immunity lasts for the time interval analyzed here.

12 During the second half of 2020 worked hours were 3.77 per cent lower than during the corresponding period in 2019.

13 The economic outcomes in year 2 after the start of vaccinations are hardly affected by the vaccination strategy. We therefore restrict our analysis to the effects during the first year of the vaccination campaign.

14 We have also simulated alternative strategies, such as all groups being vaccinated simultaneously at rates in proportion to their share of the total population. None of these strategies have generated optimal outcomes in terms of any of the outcome measures that we focus upon.

15 Sweden had a GDP of about 5000 billion SEK or 465 billion USD (at current exchange rates) in 2020.

16 As will be demonstrated in Sect. Transmission rates, a higher transmission rate will increase this difference substantially.

17 We obtain similar improvements in terms of our outcome measures when increasing the vaccination rate instead of the vaccine efficacy. For $e=0.5$ and $u=0.005$ the fatality rate is 519-620 per million people, the total share of infected persons is 15.5-17.7 per cent, the peak infection rate is 0.51–0.53 per cent and the economic gains are 1.21-1.65 per cent (to be compared with the outcomes for $e=1$ and $u=0.0025$ presented above).

18 We obtain similar improvements in terms of our outcome measures when increasing the vaccination rate instead of the vaccine efficacy. For $e=0.5$ and $u=0.005$ the fatality rate is 748–1085 per million people, the total share of infected persons is 17.9–27.3 per cent, the peak infection rate is 0.72–1.23 per cent and the economic gains are 1.14– 2.28 per cent (to be compared with the outcomes for $e=1$ and $u=0.0025$ presented above).
